# The Importance of Correlating Bedside Observation and Laboratory Methods of Diagnosis

**Published:** 1918-09

**Authors:** C. E. Durham

**Affiliations:** Hico, Texas


### The Importance of Correlating Bedside Observation and Laboratory Methods of Diagnosis.

DR. C. E. DURHAM, HICO, TEXAS.

---

The majority of mistakes in diognosis are due not to poverty of methods, but to neglect or ignorance of well established clinical and laboratory tests. So our available methods of diagnosis are not always employed. And further we may say that the easier the diagnosis, the worse the prognosis. The clinician may and often does neglect laboratory tests. The up-to-date and strictly laboratory diagnostician does not put sufficient stress on the bedside clinical investigation. And there is no doubt that in many, many instances we are handicapped, especially in the early stages of the disease, by a total or partial absence of clinical evidence and by a neglected lack of an exact laboratory method applied. Yet, I believe that every one will cordially consent that there should be a correlation between the bedside observations and the laboratory findings.

It is important that the clinician should know the history of the case, family and personal. And the majority of us fail to recognize that a clinical history which carefully records all the possible etiological factors, the symptoms at the beginning of the trouble, and the development and picture of the clinical course, as well as the present condition, is not only of added value for the diagnosis and future treatment, but, in addition, leaves a record in our hands by which we can picture this disease as a clinical entity in its beginning. The family record may have a great bearing on the prognosis of any case, for it is just as important to know what kind of a patient has a disease as it is to know what kind of a disease the patient has. The patient, when he presents himself, is chiefly impressed with the symptoms from which he suffers at the time of examination, and may not, and often does not, remember the symptoms of the beginning of the trouble. Not only this, but the mental horizon of the physician seldom extends beyond the present state of the disease. The patient, seeking help, demands relief. It is hard to put him back into the beginning of the disease, and so he must be treated as he is when he first seeks help. Yet are we doing our patient and ourselves equal justice by proceeding without investigating the family history and taking his true clinical record? Let us not forget that the more evident the symptoms and the easier the diagnosis the worse the clinical history of the early stages.

If we are to look upon the clinician as a halper to the surgeon, for most of the surgeon's work is seen by the home physician at the bedside, it might be said that the surgical technique has advanced more rapidly than surgical diagnosis. The surgeon should be able to present to the medical profession the minimum of symptoms of the early stage of surgical diseases in which the immediate and ultimate results of treatment are best. Often the family physician has not been trained to recognize the significance of the symptoms as the beginning of a surgical lesion. Then if the people seek advice earlier and the family physician from his bedside observations can properly interpret these lesser and apparently insignificant signs of future trouble, the surgeon will be called more often to treat surgical lesions in their earlier stages.

And if we take surgical diseases, when there is no danger from delay, there is opportunity to exhaust every method of diagnosis and to employ every well established preparatory treatment. The earlier the patient is seen and the less distinct the indications, the more elaborate and painstaking should be the investigation and the more careful the preparation. I believe that the evidence will show that at the present stage of our development, more mistakes are made by hurrying operative intervention in some chronic groups than by delay in some of the acute groups. My observation is especially called to that class of patients suffering from chronic appendicitis. The patient, the family physician and the surgeon arrive quickly at such a diagnosis, for the people are becoming well educated in regard to the dangers of appendicitis. The pre-operative diagnosis is usually insufficient. The appendix is removed through a small incision. The number of patients not relieved is increasing. Some other lesion is overlooked, among which may be mentioned some type of gastric and colon ptosis, some lesion of the pelvic or vaginal outlet, gastric or duodenal ulcer, chronic infection of the gall bladder, chronic pancreatitis, cancer of colon, renal calculus, etc. We are too quick to make up our minds as to what should be done and we fail to apply all of our known methods or to apply them scientifically. Successful operations are sometimes failures due to the fact that the surgeons have placed too much confidence in the bedside observations.

The next point under bedside observation is physical diagnosis. How easy it is to get into a routine method of doing as little amount of work as possible in making a physical examination. We often take too much for granted. We think that we know already too much about the patient's past, his family and his environment and so are not as careful as we should be in our investigations. In truth, I am almost persuaded that, at times, it might be best for the patient to be examined by another clinician who is not influenced by any past experience or previous knowledge of the case. If he has a particular hobby, this hobby may not fit into the other man's, so new things may be discovered. Any way, by whomsoever made, each physical examination should be thorough. Nothing should be taken for granted, or too many questions asked, for the patient will answer accordingly, thinking that he is helping you in your diagnosis, when, in fact, he is putting stumbling blocks in your way. When properly done, it is no small job to make a physical examination. There is the head, the throat, the nose, the chest, the skin, the abdomen, the heart; there are the eyes, the ears, the limbs; the blood pressure must be taken, and other things must be done. Care in each step of the examination must be taken, for something overlooked somewhere may cause a mistaken diagnosis as a summary of things wrongly seen and heard. We must be so accurate in our examination and so exacting in our history taking, that our conclusions have only to be confirmed by the laboratory and not corrected.

No matter how exact the science of medicine may become in its diagnostic methods, the art of medicine, which rests on clinical investigations and deductions, should never be neglected. At present with many of us the easier and more available laboratory methods of diagnosis are not employed as a routine, but only in special cases. The more careful we are, the more convinced we become that many of the tests should be employed as a routine procedure. But the general practitioner, the busy man, will say that he has not the time. It should be not alone from a mercenary standpoint that we practice medicine. We should have other objects in view; the advancement of our own knowledge for present and future use, and the best diagnosis for the case in hand and future cases. This being true, then we must perfect our technique in all laboratory methods. We are to do this to confirm our bedside observations which should be used independently only when we fail to demonstrate fully the cause from the bedside technique.

And again, it is important that we may be able to interpret the laboratory findings. And in practical surgery to utilize the interpretations of these laboratory methods, one must repeatedly bring together these findings before an operation and the picture found at the operation. And if we have been exact in our bedside observation and definite in our laboratory demonstration, we have sufficient available evidence which will enable the internal medical man and the surgeon ,if they have investigated these records, to know what is best to do for the patient and to prepare themselves for future responsibilities when called on to treat other lesions in their earliest stages. I know of nothing more inhibatory to the growth of a medical man than desultory, superficial, clinical examination. Often when a case is brought to him, the internist or the surgeon is handicapped or hampered by the advanced stage of the disease, owing to a failure on the part of someone to recognize the gravity of the situation. The ability to make an early diagnosis not only redounds to one's own credit and to the good of the patient, but it enhances the value of internal medicine and surgery.

The science of medicine seems to be practically without limit. There should be complete harmony existing in the work done by the internist, the laboratory specialist and the operator.

This can be done only when all strive to attain the highest efficiency in their separate spheres of activity. We must get together, stay together and work together, for before us are fields of wonderful possibilities and splendid achievements sufficient to satisfy all.

I trust that bedside observation may not become a lost art. The men who see the cases in the different homes must not rely too much upon the opinion of the man behind the microscope. I would not have you think that I minimize the work done in the laboratory. Far from it. But I do not believe that we are justified in allowing our technique to go by default because it is easier for us to get some one to do the diagnosing for us. Often when we seek the help of the pathologist we find that he is handicapped on account of his lack of familiarity with the clinical manifestations. So we may expect too much of the laboratory. Many pathologists are too certain of their frozen section diagnosis. Many clinicians and operators are often not willing to grant mistakes shown them by the pathologist.

And when we as operators, clinicians, pathologists and specialists can perfectly unify our work and strive to be the most efficient in our special line, then we can use the work of the one to demonstrate the correctness ff that of the other, and so correlate and unify the findings in general, for this great government is entitled to demand of every one of us a complete knowledge of and a profound interest in the physical life both of the young and the old.

